# Impact of multidisciplinary team management in head and neck cancer patients

**DOI:** 10.1038/bjc.2011.92

**Published:** 2011-03-29

**Authors:** P L Friedland, B Bozic, J Dewar, R Kuan, C Meyer, M Phillips

**Affiliations:** 1 Ear Sciences Centre, School of Surgery, University of Western Australia, Perth, Western Australia, Australia; 2 Department of Otolaryngology, Head, Neck and Skull Base Surgery, School of Surgery, University of Western Australia, Sir Charles Gairdner Hospital (SCGH), Nedlands, PO Box 3092, Perth 6009, Western Australia, Australia; 3 Department of Medical Oncology (SCGH), Perth, Western Australia, Australia; 4 Department of Radiation Oncology (SCGH), Perth, Western Australia, Australia; 5 Hospital Based Cancer Registry, Sir Charles Gairdner Hospital, Perth, Western Australia, Australia; 6 Western Australian Institute of Medical Research, University of Western Australia, Perth, Western Australia, Australia

**Keywords:** multidisciplinary clinic, primary head and neck cancer, survival, cancer management

## Abstract

**Background:**

We analysed the outcomes of 726 cases of primary head and neck cancer patients managed between 1996 and 2008, including those managed in the multidisciplinary clinic or team setting (MDT) and those managed outside of an MDT by individual disciplines (non-MDT) in the same institution.

**Methods:**

Data were collected from the Hospital Based Cancer Registry and a database within the Head and Neck Cancer Clinic. Univariable comparisons and multivariable analyses were performed using a logistic regression model. Survival by staging was analysed. Comparisons of management and outcomes were made between MDT and non-MDT patients.

**Results:**

395 patients (54%) had been managed in the MDT *vs* 331 patients (46%) non-MDT. MDT patients were more likely to have advanced disease (likelihood ratio *χ*^2^=44.7, *P*<0.001). Stage IV MDT patients had significantly improved 5-year survival compared with non-MDT patients (hazard ratio=0.69, 95% CI=0.51–0.88, *P*=0.004) and more synchronous chemotherapy and radiotherapy (*P*=0.004), and the non-MDT group had more radiotherapy as a single modality (*P*=0.002).

**Conclusions:**

The improved survival of MDT-managed stage IV patients probably represents both the selection of multimodality treatment and chemotherapeutic advances that these patients received in a multidisciplinary team setting by head and neck cancer specialists as opposed to cancer generalists in a non-MDT setting.

Head and neck (H&N) cancers are a complex, heterogeneous group of malignancies, which require multifaceted treatment strategies and the input of a number of specialities. To facilitate timely and appropriate evidence-based management of H&N cancer cases, most centres have now established multidisciplinary team meetings (MDT) in which each of the medical and allied health specialities are represented so that accurate tumour staging and treatment plans can be best tailored to individuals (Expert Advisory Group on Cancer, 1995; Taylor *et al*, 2010).

A challenge for service providers is the lack of level I evidence in H&N cancer management. An MDT provides a combination of evidence-based management, local experience and availability of treatment modalities. The assumed benefits of the MDT include improvements in communication between health professionals, coordination and continuity of care and better clinical outcomes (Westin and Stalfors, 2008). Despite this, MDTs are costly, and their benefits in improving outcomes in the management of H&N cancer have not been widely studied (Westin and Stalfors, 2008; Taylor *et al*, 2010).

The Sir Charles Gairdner Hospital Based Cancer Registry (HBCR) was started in 1996 for systematic collection of data on patients with certain primary cancers, including H&N malignancies. A weekly H&N Cancer MDT is attended by otolaryngologists, plastic surgeons, radiation and medical oncologists, dentists, dieticians, speech pathologists, radiologists and a nurse cancer care coordinator. The MDT reviews each new patient's diagnosis, imaging, medical and social factors, confirms staging and formulates a management plan. After initial treatment, cases are reviewed again at the MDT whenever further treatment is planned. The MDT follows the National Comprehensive Cancer Network Clinical Practice Guidelines in Oncology.

Despite the availability of the MDT at SCGH, we recognised that a large proportion of newly diagnosed patients are still managed by individual disciplines outside of the MDT, which included clinicians who were in fact generalists with an interest in H&N cancer but also clinicians who were members of the MDT.

The primary aim of our study was to analyse the differences in outcome and survival data between these two groups of H&N cancer patients managed at SCGH.

## Materials and methods

A retrospective 12-year analysis was undertaken, involving data from the SCGH HBCR and SCGH MDT. This study was approved by the Sir Charles Gairdner Human Research Ethics Committee (QI no 2444). Patients attending the MDT were entered into a Microsoft Access database and were also included in the HBCR. Newly diagnosed primary H&N cancer patients presenting to the hospital for treatment (MDT and non-MDT) from 1 January 1996 until 1 February 2009, whose complete hospital and HBCR records were obtainable, were included.

Patient demographics, cancer site, staging, treatment and outcomes of different modalities and combinations of therapies were compared. All cases were followed-up for death by the Registrar General of Western Australia. Statistical methods for analysis included Cox proportional hazards regression and likelihood ratio *χ*^2^-tests (LR*χ*^2^). Analysis of survival was used to estimate the hazard ratio (HR), with associated 95% confidence intervals and *P*-values. For all analyses, a *P*-value <0.05 was interpreted as statistically significant, but for univariable Cox regression results, the sequential rejection method of Holm was used to correct the critical significance level for multiple comparisons (Holm, 1979). Data analysis was conducted using the Stata package (StataCorp, 2009. Stata Statistical Software Release 11; StataCorp LP, College Station, TX, USA).

## Results

**Figure 1:**
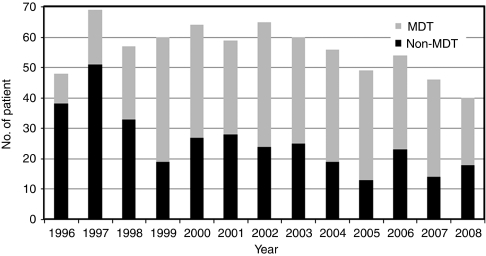
Head and neck cancer patients seen at MDT and non-MDT, 1996–2008.

**Figure 2:**
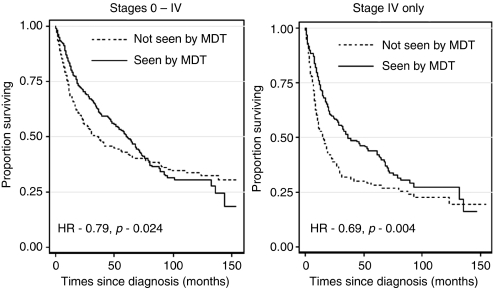
Kaplan–Meier survival curves for H&N cancer patients for all stages and Kaplan–Meier survival curves for H&N cancer patients: stage-IV cases only.

A total of 726 newly diagnosed patients fulfilled the inclusion criteria (see Figure 1). MDT patients were younger by about 2 years of age on average (*P*=0.046), which is a potential source of bias, as increasing age at diagnosis is associated with a significant increase in risk of death (HR=1.04, *P*<0.001). Survival times were further analysed for stage I–IV groups individually, and there was no significant difference in outcomes for stages I–III between MDT- and non-MDT-treated patients (see Table 1). The numbers in each of these stages were too small to provide adequate statistical power. There was, however, a statistically significant difference in survival for stage-IV patients who were managed by the MDT (HR=0.69, 95% CI=0.51–0.88, *P*=0.004; see Figure 2). Furthermore, patients seen in the multidisciplinary clinic were more likely to have advanced disease (LR*χ*^2^=44.7, *P*<0.001), which would produce bias, suggesting greater risk for that subset of patients, if stage was not controlled for in the analysis.

Analysis of therapy variables suggests that the multidisciplinary clinic has a substantial influence on treatment decisions. Patients seen in the multidisciplinary clinic were significantly less likely to receive radiotherapy alone for positive nodes (LR*χ*^2^=16.08, *P*<0.001), significantly less likely to receive surgical treatment alone for their cancer (LR*χ*^2^=10.74, *P*<0.001) and positive nodes (LR*χ*^2^=19.42, *P*<0.0001). There was an increasing incidence in the use of combination chemotherapy and radiotherapy from 12.5% in 1996 to 45% in 2008 (Cuzick test for trend, *P*<0.001; Cuzick, 1985) and a concomitant decline in the use of radiotherapy alone from 27.1% in 1996 to 15% in 2008 (Cuzick test for trend, *P*<0.001). Synchronous chemotherapy and radiotherapy increased from 2.1% in 1996 to 42.5% in 2008 (Cuzick test for trend, *P*<0.001). Combination surgery, chemotherapy and radiotherapy increased from 6.3% in 1996 to 12.5% in 2008. There was a clear difference in treatment modalities in the MDT *vs* non-MDT group. The MDT has more synchronous chemotherapy and radiotherapy (*P*=0.004) and the non-MDT group has significantly more radiotherapy as a single modality (*P*=0.002).

## Discussion

Despite widespread implementation of MDTs in cancer management in a number of countries, robust evidence to suggest improvement in outcomes in patients with H&N cancer is lacking, which contributes to scepticism regarding MDT patient management. The literature suggests that MDT time delays and expense are some potential reasons why treating doctors believe that early-stage H&N cancers can be successfully managed outside of an MDT, principally referring patients with advanced malignancy (Taylor and Ramirez, 2009). Apart from influencing treatment decisions, MDTs have also been shown to improve cancer staging and subsequently patient outcomes (Stephens *et al*, 2006). Consequences of non-MDT management include the possibility of less accurate staging, lack of allied health input and loss to follow-up, due to the lack of coordinated care, as is available in MDT settings. A search of the medical literature reveals a number of cohort studies from single centres or regions that have demonstrated survival benefits linking MDT management in a range of malignancies. (Junor *et al*, 1994; Birchall *et al*, 2004; Morris *et al*, 2006; Stephens *et al*, 2006). However, they are subject to bias and therefore it is difficult to say whether results from one centre can be extrapolated to another centre (Expert Advisory Group on Cancer, 1995). Nevertheless, in the absence of level I or II data, these studies form the basis of clinical guideline formulation and inform current best practice.

A surprisingly high proportion of H&N cancer patients at SCGH were managed independently of the MDT by various disciplines, including otolaryngology, plastic surgery, general surgery, radiation oncology and medical oncology. This presented a unique opportunity to analyse the potential differences in management and outcomes between the two cohorts of patients. Investigating the reasons why some of our colleagues never referred or inconsistently referred all H&N cancer patients to the MDT was beyond the scope of this study. However, we do acknowledge that this may be a potential source of bias in reporting our results.

Our results indicate two principal findings. First, there is a significant increase in survival for patients managed through the MDT when stage, age at diagnosis and year of diagnosis are controlled for in the analysis. Second, the use of a selection of multimodality therapy treatment options is significantly associated with management of patients by the MDT and it seems likely that this is also the cause of the reduced risk of death for MDT. These findings may be explained by the fact that H&N cancer specialists (MDT) as opposed to cancer generalists (non-MDT) are involved in management and that a large proportion of the non-MDT patients were treated more than 10 years ago when advances in chemotherapeutic therapies were not yet present. The adoption of these recent chemotherapeutic advances in the MDT may account for increased survival of stage-IV MDT-treated patients.

The results of this study have been openly discussed with all our colleagues in our institution and we have recommended adherence to evidence-based guidelines and management of all H&N cancer cases in an MDT irrespective of staging. In the last 12 months since the release and discussion of these findings, opinions and viewpoints regarding the H&N cancer MDT have shifted to an increase in patient referrals of all cancer stages. A repeat audit of patient outcomes in the future is recommended.

## Figures and Tables

**Table 1:** H&N cancers, 1996–2008

	**Seen at MDT**		**Difference between MDT and non-MDT**	**Difference in survival**
	**No (%)**	**Yes (%)**	***P***	***P***
*Tumour site*				
Oral cavity	49 (14.8)	92 (23.3)	0.003	0.761
Oropharynx	74 (22.3)	116 (29.4)	0.03	0.665
Nasopharynx	17 (5.12)	11 (2.78)	0.103	0.044
Hypopharynx	16 (4.82)	13 (3.29)	0.295	<0.001
Larynx	93 (28.0)	90 (22.8)	0.106	0.048
Nasal cavity/sinus	42 (12.7)	39 (9.87)	0.237	0.01
Salivary glands	36 (10.8)	25 (6.33)	0.029	0.012
Other	5 (1.51)	9 (2.28)	0.446	0.174
Total	332 (100)	395 (100)		
				
*Treatment modality*			<0.001	
Chemotherapy only	2 (0.87)	2 (0.76)	0.862	0.009
Surgery only	31 (13.4)	42 (16.0)	0.562	0.08
Radiotherapy only	120 (52.0)	83 (31.7)	<0.001	0.028
Radiotherapy+ chemotherapy	27 (11.7)	28 (10.7)	0.002	0.572
Radiotherapy+ chemotherapy+surgery	16 (6.9)	18 (6.9)	0.868	0.189
Synchronous chemotherapy	35 (15.2)	89 (34.0)	<0.001	0.039
Total	231 (100)	262 (100)		

Abbreviations: H&N=head and neck; MDT=multidisciplinary team.

Grouping according to the UICC (Union for International Cancer Control) TNM version 6 and treatment pattern overall.
